# Attention-based deep residual learning network for entity relation extraction in Chinese EMRs

**DOI:** 10.1186/s12911-019-0769-0

**Published:** 2019-04-09

**Authors:** Zhichang Zhang, Tong Zhou, Yu Zhang, Yali Pang

**Affiliations:** College of Computer Science and Engineering,Northwest Normal University, 967 Anning East Road, Lanzhou, 730070 China

**Keywords:** Chinese electronic medical record, Entity relation extraction, Deep residual learning network, Attention mechanism

## Abstract

**Background:**

Electronic medical records (EMRs) contain a variety of valuable medical concepts and relations. The ability to recognize relations between medical concepts described in EMRs enables the automatic processing of clinical texts, resulting in an improved quality of health-related data analysis. Driven by the 2010 i2b2/VA Challenge Evaluation, the relation recognition problem in EMRs has been studied by many researchers to address this important aspect of EMR information extraction.

**Methods:**

This paper proposes an Attention-Based Deep Residual Network (ResNet) model to recognize medical concept relations in Chinese EMRs.

**Results:**

Our model achieves *F*_1_-score of 77.80% on the manually annotated Chinese EMRs corpus and outperforms the state-of-the-art approaches.

**Conclusion:**

The residual network-based model can reduce the negative impact of corpus noise to parameter learning, and the combination of character position attention mechanism will enhance the identification features of different type of entities.

## Background

EMR is used by medical staff to record texts, symbols, charts, graphics, data, and other digital information generated by HIS (hospital information system). With the tremendous growth of the adoption of EMR, various sources of clinical information (including demographics, diagnostic history, medications, laboratory test results, and vital signs) are becoming available, which has established EMR as a treasure trove for large-scale analysis of health data. Unstructured medical text in EMR is one kind of narrative data, including clinical notes, surgical records, discharge records, radiology reports, and pathology reports. For the convenience of narration, we use EMR to represent unstructured EMR text in the following.

Identifying semantic relations existing among medical concepts in EMRs is of great importance to health-related various applications. These relations are hold between medical problems, tests, and treatments. Table 1 presents two examples of semantic relation, one of which is between medical concept *e*_1_=“*cold*” and *e*_2_=“*fever*” in sentence *S*_1_, and the other is between *e*_1_=“*Head MRI*” and *e*_2_=“*lacunar infarction*” in sentence *S*_2_.

**Table 1:** Examples of the relations between medical entities

Sentence	Relation
*S*_1_: The patient has a *cold*, feels a*fever* and headache.	Disease causes symptoms (DCS)
*S*_2_: *Head MRI* shows *lacunar infarction*.	Test reveals the disease (TeRD)

On account of the importance of this subject, the 2010 i2b2/VA NLP challenge for clinical Records presented a relation classification task focused on assigning relation types between medical concepts in EMRs. Since then medical concept relation classification has being paid attention by more and more researchers.

In the traditional natural language processing (NLP) research, semantic relations between named entities can be used for many applications including knowledge graph construction, sentiment analysis, question answering, etc. [1], relation extraction or classification therefore has always been an important issue [2]. In previous open-domain entity relation extraction studies, researchers applied many different traditional machine learning models include Logistic Regression, SVM and CRF to recognize relations [3–7]. Li et al. used CRF model to reduce the space of possible label sequences and introducing long range features for relation recognition [8]. Mintz et al. put forward a remote monitoring relation classification method which could generate adequate training data by aligning text and knowledge base to solve the problem of lack of enough training data [9]. Socher et al. firstly employed recurrent neural network (RNN) on the task of relation extraction, while utilizing the syntactic structure information of sentences [10]. Miwa et al. proposed a neural network relation extraction architecture based on bidirectional LSTM and tree LSTM to encode entities and sentences simultaneously [11].

Drawing on these studies on open-domain relation extraction, similar task on EMRs was formally defined in the 2010 i2b2/VA Challenge Evaluation [12]. Some researchers proposed various models for relation classification of EMRs. Bruijn et al. used SVM to train multiple classifiers to deal with different relation categories, and improved the effect of classification [13]. Rink et al. use external dictionaries to increase the effect of entity relationship recognition [14]. Fang et al. extracted the relations from relevant articles of Chinese herbal medicine based on manually designed rules and created a relation database [15]. Zhou et al. utilized a bootstrapping framework to extract relations from the medical articles and created a knowledge base [16]. Li et al. raised an electronic health records relation classification model based on CNN-LSTM [17]. Overall, the existing models mainly focus on English EMR texts, and on the other hand it still cannot deliver satisfactory recognition performance. Concerning the increasing availability of digitalized Chinese EMRs, this paper addresses the semantic relation identification problem among medical concepts in Chinese EMRs. We propose an attention mechanism based deep residual network model to classify the medical entity relations in Chinese EMRs. Experimental results performed on a manually labeled Chinese EMR corpus show that our model achieved better performance with *F*_1_-score of 77.80% compared with other methods.

## Methods

**Fig. 1:**
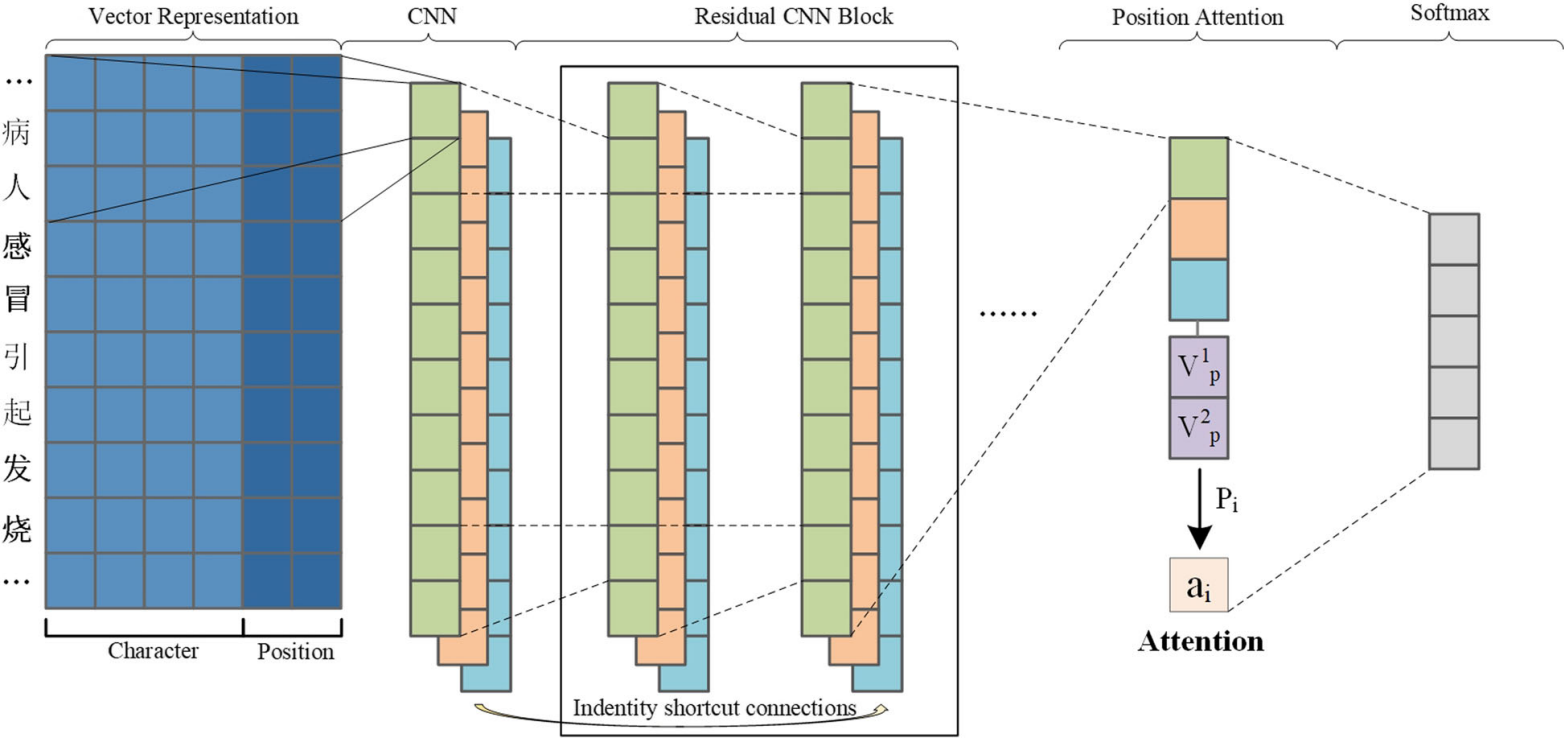
The architecture of our relation extraction model

Our model is based on a CNN architecture as shown Fig. 1. The model consists of five parts: vector representation layer, convolution layer, residual networks layer, position attention layer and output layer.

### Character embedding

Given a Chinese sentence *S*=(*c*_1_,*c*_2_,…,*c*_*n*_) which contains two entities *e*_1_ and *e*_2_. Each character *c*_*i*_ will be mapped to a low-dimensional dense vector $V_{i} = (V_{w}^{i}, V_{p}^{i})$, in which $V_{w}^{i}$ represents the character vector and $V_{p}^{i}$ is the vector of character position in the sentence. The character embedding initialized with vector which is pre-trained by word2vec, and *d*_*w*_ is the dimension of character vector.

### Position embedding

**Fig. 2:**
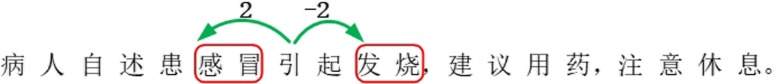
An example of the relative distance between an entity and a character. The relative distance of a character to medical entity “(*cold*)” and “(*fever*)” are 2 and -2 respectively

Position embedding $V_{p}^{i}$ is also a low-dimensional vector of character position in the sentence, which can combine the relative positions (see Fig. 2) of the current character to the first entity *e*_1_ as well as the second entity *e*_2_. Each relative position corresponds to a position embedding $V_{p}^{i} \in R^{d_{p}}$, *d*_*p*_ is the dimension of position embedding.

The vector $\phantom {\dot {i}\!}V_{i} \in R^{d_{v}}$ is concatenation of character vector $V_{w}^{i}$ and two position vectors, where *d*_*v*_=*d*_*w*_+2*d*_*p*_.

### Convolution

Convolution is to extract the effective local feature information from characters and their corresponding contexts. The *V*_*j*_ is a vector which corresponds the *j*-th character in the sentence *S*=(*V*_1_,*V*_2_,…,*V*_*n*_), here *n* is the sentence length. We use filter $\phantom {\dot {i}\!}W \in R^{h \times d_{v}}$ to extract local features from the sentence *S*. A feature *c*_*j*_ is generated from a window of character *V*_*j*:*j*+*h*−1_ by

1 $$ c_{j}=f(W \cdot V_{j:j+h-1}+b),  $$

where *b* is a bias terms and *f* is a non-linear function. We apply dropout layer in convolution to prevent data from outfitting.

### Residual networks

**Fig. 3:**
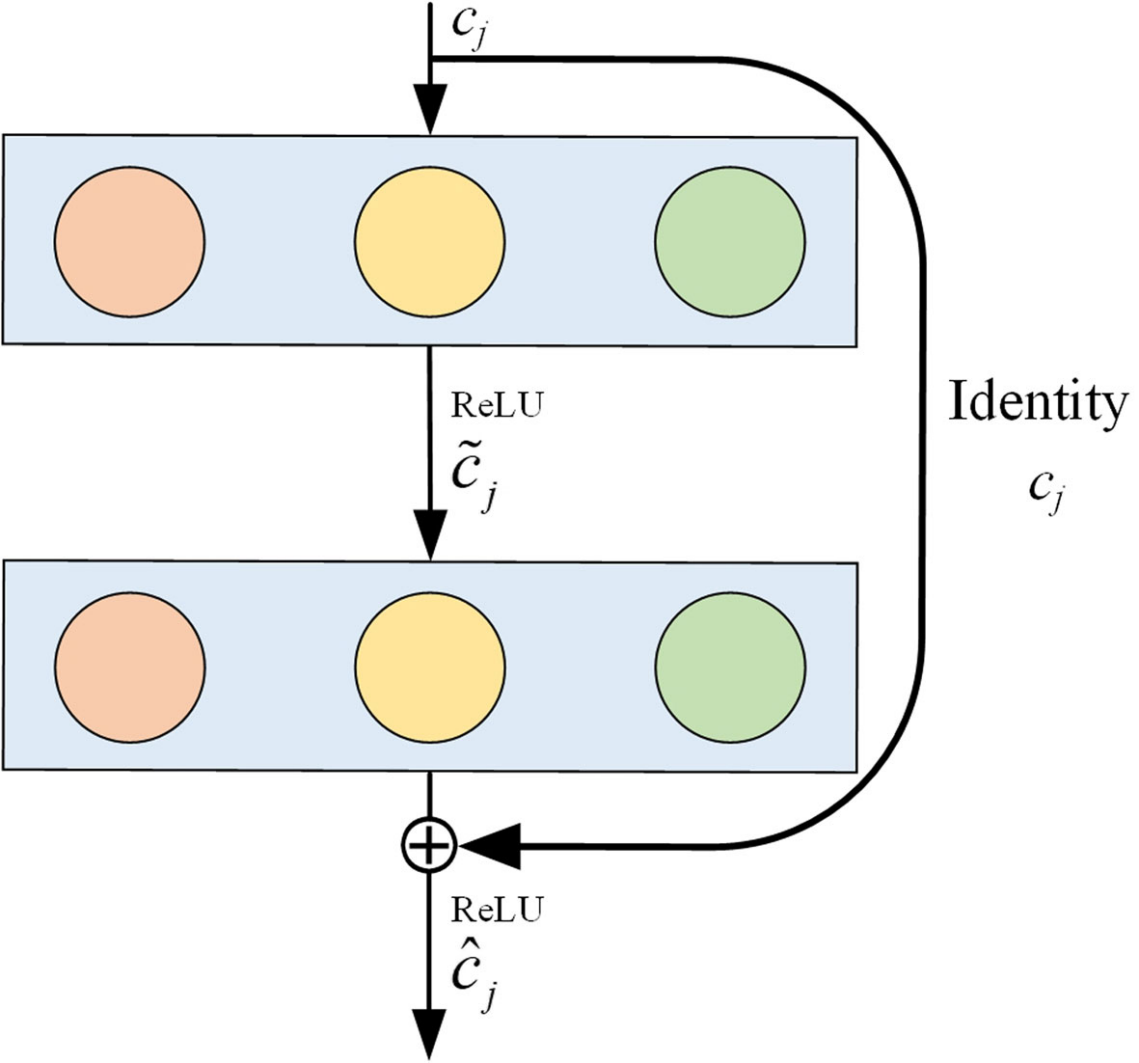
The residual convolution block

Residual learning connects low-level to high-level representations directly and solves the vanishing gradient problem, we superimposed the identity mapping function on a network. In our model, each residual convolution block (see Fig. 3) has two convolutional layers, each one followed by a ReLU activation, we use shortcut connection between each of the residue convolution block *W*_1_,*W*_2_∈*R*^*h*×1^ are two convolution filters, where h is convolution kernel size. The first convolutional layer is

2 $$ {\tilde c_{j}} = f\left(W_{1} \cdot c_{j:j + h - 1} + b_{1}\right),  $$

and the second is

3 $$ \hat c_{j} = f\left(W_{2} \cdot \tilde c_{j:j + h - 1} + b_{2} + c_{j}\right),  $$

here *b*_1_, *b*_2_ are bias terms. The residual convolution block output is the vector $\hat c_{j}$. This block will be multiply concatenated in our architecture by a shortcut connection.

### Position attention

Recently attention mechanism has been widely used in machine learning, and great achievements have been made in various NLP problems. In this paper, we use the position attention to enhance relation extraction ability. Firstly, we carry the max-pooling operation on the residual learning result. Secondly, as shown in Fig. 1, we concatenate the max-pooling results with the position embedding of entity. Finally, we use the attention mechanism to balance the weight to the sentence.

4 $$ S_{i} = \sum\limits_{i} \alpha_{i} \times P_{i},  $$

where *α*_*i*_ represents the attention weight. *P*_*i*_ is a result which concatenates the max-pooling results with the position embedding of entity. Finally, we use the softmax function to normalize and output entity relation probability.

5 $$\begin{array}{*{20}l} \alpha_{i} = \frac{\exp \left(e_{j}^{i}\right)}{{\sum\nolimits}_{k} {\exp \left(e_{j}^{k}\right)}} \end{array} $$

## Results

### Dataset and evaluation metrics

On the basis of reference to medical semantic relation annotation specification of 2010 i2b2/VA Challenge, we established our own relation annotation specification of Chinese EMRs, in which semantic relations between medical concepts fall into five coarse-grained categories and fifteen fine-grained categories. All of relation category are detailed as follows.

*Coarse-grained category 1:* Treatment -Disease Relation. This category contains five fine-grained categories, including TrID (Treatment improves the disease), TrWD (Treatment worsens the disease), TrCD (Treatment causes the disease), TrAD (Treatment is administered for the disease), and TrNAD (Treatment is not administered because of the disease).

*Coarse-grained category 2:* Treatment -Symptoms Relation. This category also contains five fine-grained categories, including TrIS (Treatment improves the symptoms), TrWS (Treatment worsens the symptoms), TrCS (Treatment causes the symptoms), TrAS (Treatment is administered for the symptoms), and TrNAS (Treatment is not administered because of the symptoms).

*Coarse-grained category 3:* Test-Disease Relation. This category contains two fine-grained categories, including TeRD (Test reveals the disease) and TeCD (Test conducted to investigate the disease).

*Coarse-grained category 4:* Test-Symptoms Relation. This category also contains two fine-grained categories, including TeRS (Test reveals the symptoms) and TeBS (Test based on symptoms).

*Coarse-grained category 5:* Disease-Symptoms Relation. This category contains only one fine-grained category named as DCS (Disease causes symptoms).

According to our specification, we manually annotated 3000 de-identified Chinese EMR texts from different clinical departments of a grade-A hospital of second class in Gansu Province, China. 2000 medical texts are selected as training data, 500 medical texts as develop data, and 500 medical texts for test while evaluating our method on this dataset. The relation numbers of every fine-grained category in this dataset are given in Table 2. *Precision*, *Recall* and *F*_1_*-score* are used as evaluation metrics.

**Table 2:** The relation number of every fine-grained category in the corpus

Fine-grained category	Traing	Develop	Test
TrID	368	260	193
TrWD	229	149	102
TrCD	423	284	265
TrAD	4706	2096	1581
TrNAD	110	35	41
TrIS	1351	371	427
TrWS	598	152	163
TrCS	118	41	57
TrAS	2093	1083	1154
TrNAS	98	36	21
TeRD	1770	498	603
TeCD	85	23	27
TeRS	13963	8180	4998
TeBS	1492	214	388
DCS	5465	2677	3251

### Models and parameters

We carry out the experiments to compare the performance of our model with others described in the following.

***CNN-Max***: This model was used by Sahu, et al. [18], which encoded the sentence vectors with CNN, and outputted the results after max-pooling and softmax function.

***BLSTM-Attention***: This model was proposed by Li, et al. It mainly consists of bidirectional LSTM and attention mechanism [19].

***ResNet-Max***: This model was proposed by Huang, et al. Compared with our model, this model did not combined attention mechanism [20].

***ResNet-BLSTM***: The basic framework of the method is close to our model. The difference between this one with ours is that this model combine the residual network with Bi-LSTM.

***ResNet-PAtt***: This is the model presented in this paper. Table 3 gives the chosen hyper-parameters for all experiments. We tune the hyper-parameters on the development set by random search. We try to share as many hyper-parameters as possible in experiments.

**Table 3:** Hyper parameters of the residual neural network

Parameter	Description	Value
*d*_*w*_	Dimension of word embedding	100
*d*_*p*_	Dimension of position embedding	5
*k*	Window size	3
*m*	Number of filters	128
*B*	Batch size	50
*λ*	Learning rate	0.01
*p*	The ration of dropout	0.3

### Experimental results

Table 4 shows the overall classification performance of different models on our evaluation corpus. It can be seen that our method ResNet-PAtt is better than other methods in *F*_1_-score while precision, recall and *F*_1_-score reaches 79.16 and 77.80% respectively. Of all other methods, the model ResNet-BLSTM achieves the best performance on *F*_1_-score, and our model improves 2.97% *F*_1_-score compared with it, then our method is more effective. In addition, we can find that overall the residual network based methods are better than other relation extraction methods.

**Table 4:** Comparison of overall relation classification result of different model

Model	Precision	Recall	*F*_1_-score
SVM	65.24	55.26	59.84
CNN-Max [18]	55.34	50.84	52.99
LSTM-Max	69.27	70.51	69.88
BLSTM-Attention [19]	74.12	66.95	70.35
ResNet-BLSTM	**78.81**	71.24	74.83
ResNet-Max [20]	65.24	67.45	66.33
**ResNet-PAtt**	76.48	**79.16**	**77.80**

## Discussion

**Fig. 4:**
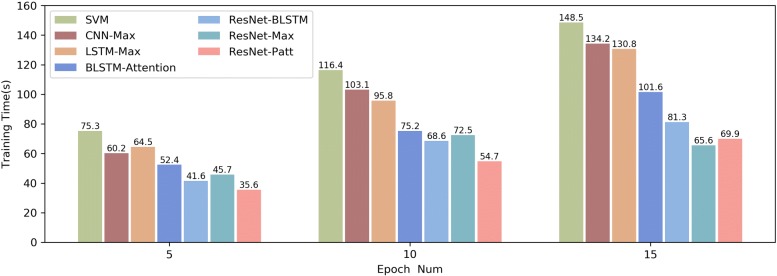
Comparison of the training time for different model

The reasons our model achieves best performance maybe owe to that the residual network-based model could reduce the negative impact of corpus noise to parameter learning, and the combination of character position attention mechanism could enhance the identification information of different type of entities. Table 5 gives the classification performance of our model on every fine-grained relation category. As can be seen from these data, our model performs best on relation category TeRS and worst on category TrNAS, which shows that it is more difficult to recognize category TrNAS correctly. We also evaluate the training time of different models. Figure 4 shows that the consumed times by these models while epoch is set as 5, 10 and 20 respectively. Overall, our model takes the shortest time to complete parameter training, and the traditional machine learning method SVM takes the longest time to train.

**Table 5:** Classification performance of our model on every fine-grained relation category.

Relation	Precision	Recall	*F*_1_-score
TrID	46.84	42.61	44.62
TrWD	41.35	40.12	40.73
TrCD	47.73	45.33	46.50
TrAD	72.42	68.48	70.39
TrNAD	45.88	46.18	46.03
TrIS	57.42	55.67	56.53
TrWS	50.21	48.28	49.23
TrCS	38.36	42.64	40.39
TrAS	61.38	80.55	69.67
TrNAS	35.76	36.51	36.13
TeRD	74.81	72.51	73.64
TeCD	41.55	39.35	40.42
TeRS	83.57	81.68	83.61
TeBS	56.72	58.31	57.50
DCS	76.86	74.53	75.68

Table 6 is comparison of *F*_1_-score for each model on every fine-grained relation category. The model has better classification performance and faster response speed.

**Table 6:** Comparison of *F*_1_-score for each model on every fine-grained relation category

Category	SVM	CNN-Max	LSTM-Max	BLSTM-Attention	ResNet-BLSTM	ResNet-Max	ResNet-PAtt
TrID	20.06	29.68	36.78	40.38	42.67	35.42	44.62
TrWD	19.35	28.34	25.35	35.21	33.21	30.43	40.73
TrCD	28.52	28.02	39.41	46.32	48.57	42.6	46.50
TrAD	63.21	43.52	58.31	71.65	68.33	64.54	70.39
TrNAD	12.36	22.46	18.24	36.69	37.26	35.42	46.03
TrIS	57.24	48.52	49.31	54.37	52.44	52.31	56.53
TrWS	36.41	49.51	37.53	46.21	48.18	42.43	49.23
TrCS	39.04	39.53	41.52	39.46	40.93	39.5	40.39
TrAS	60.26	58.33	62.34	66.83	72.36	61.36	69.67
TrNAS	13.54	14.39	14.56	28.67	30.31	24.67	36.13
TeRD	62.35	60.27	62.24	71.36	74.22	69.96	73.64
TeCD	12.34	16.52	18.36	37.23	32.88	31.48	40.42
TeRS	82.53	71.26	75.34	80.44	81.63	78.45	83.61
TeBS	48.42	46.34	47.21	58.64	57.94	51.20	57.50
DCS	64.28	65.31	65.67	74.24	73.55	70.69	75.68

## Conclusions

In this paper, we propose a deep residual network model based on the attention mechanism to classify the relation of entity pairs in Chinese EMRs. The method reduced the influence of data noise on the model training, and enhance entity discrimination feature with position attention mechanism so that the entity information can be combined effectively in the relation extraction. Experimental results show that the model reached 77.80% *F*_1_-score value, and significantly improved the classification performance of the few instance categories. At present, most relation classifications are based on entity recognition tasks and need to specify the entity in the sentence. In the future, we will study the joint extraction of entity and entity relation to further improve the efficiency of entity and entity relation recognition simultaneously.

## References

[1] Lin Y, Liu Z, Sun M (2017). Neural relation extraction with multi-lingual attention. Proceedings of the 55th Annual Meeting of the Association for Computational Linguistics:Long Papers-Volume 1.

[2] Zheng S, Wang F, Bao H, et al.Joint extraction of entities and relations based on a novel tagging scheme. 2017. arXiv preprint arXiv:1706.05075 [cs.CL]. https://arxiv.org/abs/1706.05075.

[3] Takamatsu S, Sato I, Nakagawa H (2012). Reducing wrong labels in distant supervision for relation extraction. Proceedings of the 50th Annual Meeting of the Association for Computational Linguistics: Long Papers-Volume 1.

[4] Miller S, Fox H, Ramshaw L, Weischedel R (2000). A novel use of statistical parsing to extract information from text. Proceedings of the 1st North American chapter of the Association for Computational Linguistics conference.

[5] Kambhatla N (2004). Combining lexical, syntactic, and semantic features with maximum entropy models for extracting relations. Proceedings of the ACL 2004 on Interactive poster and demonstration sessions.

[6] Culotta A, McCallum A, Betz J. Integrating probabilistic extraction models and data mining to discover relations and patterns in text. In: Proceedings of the main conference on Human Language Technology Conference of the North American Chapter of the Association of Computational Linguistics: 2006. p. 296–303.

[7] Wang T, Li Y, Bontcheva K, Cunningham H, Wang J (2006). Automatic extraction of hierarchical relations from text. Eur Semantic Web Conf.

[8] Li Y, Jiang J, Chieu H, Chai K. Extracting relation descriptors with conditional random fields. In: Proceedings of 5th International Joint Conference on Natural Language Processing. Chiang Maii: Asian Federation of Natural Language Processing: 2011. p. 392–400.

[9] Mintz M, Bills S, Snow R, Jurafsky D (2009). Distant supervision for relation extraction without labeled data. Proceedings of the Joint Conference of the 47th Annual Meeting of the ACL and the 4th International Joint Conference on Natural Language Processing of the AFNLP: Volume 2.

[10] Socher R, Huval B, Manning C, Ng A (2012). Semantic compositionality through recursive matrix-vector spaces. Proceedings of the 2012 joint conference on empirical methods in natural language processing and computational natural language learning.

[11] Miwa M, Bansal M. End-to-end relation extraction using LSTMs on sequences and tree structures. 2016. arXiv preprint arXiv:1601.00770 [cs.CL]. https://arxiv.org/abs/1601.00770.

[12] Uzuner Ö., South B, Shen S, DuVall S (2011). 2010 i2b2/VA challenge on concepts, assertions, and relations in clinical text. J Am Med Inform Assoc.

[13] De B, Cherry C, Kiritchenko S, Martin J, Zhu X (2011). Machine-learned solutions for three stages of clinical information extraction: the state of the art at i2b2 2010. J Am Med Inform Assoc.

[14] Rink B, Harabagiu S, Roberts K (2011). Automatic extraction of relations between medical concepts in clinical texts. J Am Med Inform Assoc.

[15] Fang Y, Huang H, Chen H, Juan H (2008). TCMGeneDIT: a database for associated traditional Chinese medicine, gene and disease information using text mining. BMC Complement Alternat Med.

[16] Zhou X, Liu B, Wu Z, Feng Y (2007). Integrative mining of traditional Chinese medicine literature and MEDLINE for functional gene networks. Artif Intell Med.

[17] Li L, Zhao S (2017). Relation Classification in Electronic Health Records via CNN-LSTM Model. Proceedings of the 3th China Health Information Processing Conference.

[18] Sahu S, Anand A, Oruganty K, Gattu M. Relation extraction from clinical texts using domain invariant convolutional neural network. 2016. arXiv preprint arXiv:1606.09370 [cs.CL]. https://arxiv.org/abs/1606.09370.

[19] Li L, Nie Y, Han W, Huang J (2017). A Multi-attention-Based Bidirectional Long Short-Term Memory Network for Relation Extraction. Int Conf Neural Inf Process.

[20] Huang Y, Wang W. Deep Residual Learning for Weakly-Supervised Relation Extraction. 2017. arXiv preprint arXiv:1707.08866 [cs.CL]. https://arxiv.org/abs/1707.08866.

